# Early Mental Cases

**Published:** 1902-07-26

**Authors:** 


### EARLY MENTAL CASES.

Among the more urgent problems of the moment in regard to hospital administration is the question whether or not to establish wards in general hospitals for the treatment of mental cases. We need not here repeat what we have said so often before about the pitiable and helpless position of the patient whose mental balance is beginning to be disturbed. No prudent man will certify him as insane, so into an asylum he cannot go, yet unlicensed institutions will not receive him lest they should be accused of breaking the law. Hospitals, also, generally refuse such cases, sometimes from fear of their disturbing the other patients, often because their rules specially exclude them from their benefits, thus there is in general no place left except the workhouse; and there many of these early mental cases have perforce to be received, notwithstanding the fact that they are often quite outside the pauper class. The hardship of the present state of affairs is all the greater in that a large proportion of these attacks blow over in a comparatively short space of time. Dr. Toogood,[1] medical superintendent of the Lewisham Workhouse, has recently pointed out that a very large proportion of what may be called "early mental cases" recover their mental balance within a few days under suitable treatment. Probably alcoholic excess is responsible for the great bulk of such attacks of temporary insanity. These are not cases of delirium tremens. There are many cases which give none of the signs of delirium tremens but yet are none the less certainly due to alcohol. The most common condition is that of dementia varying in degree, and these cases usually clear up within a month or six weeks. There is also a class which is distinctly maniacal and another the members of which are typically melancholic. Yet they are due to alcohol, and they clear up in a comparatively short space of time. In order to avoid sending such cases to asylums the Lewisham guardians have permitted Dr. Toogood to treat all cases of insanity in the wards of the infirmary. Taking advantage of the section of the Lunacy Act, 1890, which gives power to detain a lunatic in the wards of a workhouse for 14 days, it has become a routine never to send a case to an asylum until that period has elapsed. The results, he says, "have been remarkable—only one-third of the total number have been sent to asylums." For three years these cases were treated in ordinary small wards, there were no padded rooms, and mechanical restraint was very rarely necessary. Now there is a separate building, specially built for the treatment of mental cases, and there are padded rooms, but it is very rarely found necessary to use them. As Dr. Toogood says, surely what has been done in a poor-law infirmary can be done in a general hospital, and there is an ample field amongst the non-pauper classes who very properly object to a poor-law infirmary but would have no objection to enter the wards of a hospital. To the students the advantages would be very great, for although it would be impossible in wards devoted to "early cases" to demonstrate what Dr. Mercier calls the "varieties" of insanity, students would in such wards have a very fair opportunity of observing many of the "forms" in which insanity shows itself.

[1] Lancet, July 12.

